# Translation and Validation of the Greek Version of the Revised Fibromyalgia Impact Questionnaire (FIQR)

**DOI:** 10.31138/mjr.190525.ria

**Published:** 2026-03-01

**Authors:** Arriana Gkouvi, Eleni C. Pardali, Katerina Maria Kontouli, Eleni Patrikiou, Dimitrios P. Bogdanos, Dimitrios G. Goulis, Maria G. Grammatikopoulou

**Affiliations:** 1 Immunonutrition Unit, Department of Rheumatology and Clinical Immunology, Faculty of Medicine, School of Health Sciences, University of Thessaly, Biopolis, Larissa, Greece;; 2 Laboratory of Hygiene and Epidemiology, Faculty of Medicine, University of Thessaly, Larissa, Greece;; 3 Department of Primary Education, School of Education, University of Ioannina, Ioannina, Greece;; 4 Unit of Reproductive Endocrinology, 1^st^ Department of Obstetrics and Gynaecology, Medical School, Aristotle University of Thessaloniki, Thessaloniki, Greece

**Keywords:** fibromyalgia, Revised Fibromyalgia Impact Questionnaire, FIQR, validation, pain

## Abstract

**Objective:**

Increasing awareness of chronic pain conditions, such as fibromyalgia, requires validated tools to monitor the impact of the disease and response to treatment. This study aimed to translate and validate the Revised Fibromyalgia Impact Questionnaire (FIQR) in Greek patients with fibromyalgia.

**Methods:**

The FIQR was translated into Greek (FIQR-GR) using the forward-backward-forward method. A total of 311 adults participated in this validation study by completing questionnaires and reporting their demographic characteristics (age, gender, weight, height, education, profession, and number of children). Internal consistency was evaluated using Cronbach’s alpha, and confirmatory factor analysis (CFA) was conducted to assess the structural validity of the tool. Regression models were used to test the association between FIQR and demographic characteristics.

**Results:**

The FIQR-GR demonstrated excellent internal consistency (Cronbach’s alpha=0.94, function domain 0.92, Overall Impact domain 0.85, symptoms domain 0.85). CFA revealed goodness of fit (CFI=0.900, RMSEA=0.080, SRMR=0.056) and confirmed the original three-factor model. Most items had high standardised factor loadings (0.51 to 0.86), providing strong evidence for validity, although two items had lower values than expected (0.40 for energy levels, 0.48 for depression). There was a significant positive association between FIQR and body mass index (BMI) (p<0.001), suggesting that patients with a higher BMI experienced more severe fibromyalgia symptoms.

**Conclusion:**

The FIQR-GR is a valid and reliable tool for Greek patients with fibromyalgia and a valuable tool in clinical and research settings.

## INTRODUCTION

Fibromyalgia is a chronic disease characterised by widespread pain, sleep disturbances, fatigue, memory loss and multiple somatic symptoms.^1^ It affects 2–3% of the general population and significantly impairs quality of life.^2^ The prevalence is higher in specific patient groups such as those with type 2 diabetes mellitus or Behçet’s disease, reaching up to 80%.^3^ The intensity of symptoms can vary and is often unpredictable, which can be debilitating and disabling for the affected individual.^4^ As no definite diagnostic test is available, the American College of Rheumatology (ACR) criteria presented in the year 1990 and revised in 2010, 2011 and 2016 constitute a valuable tool for proper diagnosis.^5–8^

Fibromyalgia is often considered an invisible disease with a subjective nature, leading patients to face a dual burden: the struggle with unpredictable pain and the weight of being doubted by others.^9^ Taking into consideration the debilitating symptoms and the fluctuating disease pattern, adequately validated tools to assess fibromyalgia impact and severity are essential. Burckhardt et al*.* developed the Fibromyalgia Impact Questionnaire (FIQ), aiming to assess not only pain, but also restless sleep, fatigue, stiffness, anxiety and depression.^10^ Due to limitations associated with complex scoring and sociocultural factors, Bennett and associates developed and validated the Revised Fibromyalgia Impact Questionnaire (FIQR) in the year 2009.^11^ The FIQR is increasingly used in clinical trials and aligns with the core domains recommended by the Outcome Measures in Rheumatology Clinical Trials (OMERACT) initiative.^12^ It has been translated and validated in many languages, including Arabic, Spanish, Portuguese, Chinese, Nepali and Danish.^4,13–17^ To the best of our knowledge, translation and validation of the FIQR has not been previously performed in the Greek language. The present study aimed to translate and validate the FIQR questionnaire using a large representative sample of Greek individuals with fibromyalgia.

## MATERIALS AND METHODS

### FIQR components

The FIQR is organised into three domains, namely function, overall impact and symptoms, with a total of 21 questions. Each question assessed the severity of symptoms over the last seven days, based on an 11-point Likert scale ranging from 0 to 10, with 10 being the “worst”. The total score was calculated as follows: the sum of the first domain (range 0–90) was divided by three, the sum of the second domain (range 0–20) remained unchanged, and the sum of the third domain (range 0–100) was divided by two; these values were added together to produce the final score. The maximum score is 100.^11^ A greater score denotes more severe disease impact, while a FIQR score above 58 has been shown to exceed the Patient Acceptable Symptom State (PASS).^18^

### Translation

For the translation and cultural adaptation of the FIQR, the guidelines proposed by Guillemin et al. and the Principles of Good Practice for the translation and Cultural Adaptation of patient-reported outcomes were followed.^19,20^ Permission for the usage and translation of the FIQR was obtained from the MAPI Research Trust.^11^ An expert committee, including experienced bilingual English and Greek speakers (DPB, DGG), was formed.

**Supplementary Figure 1.**
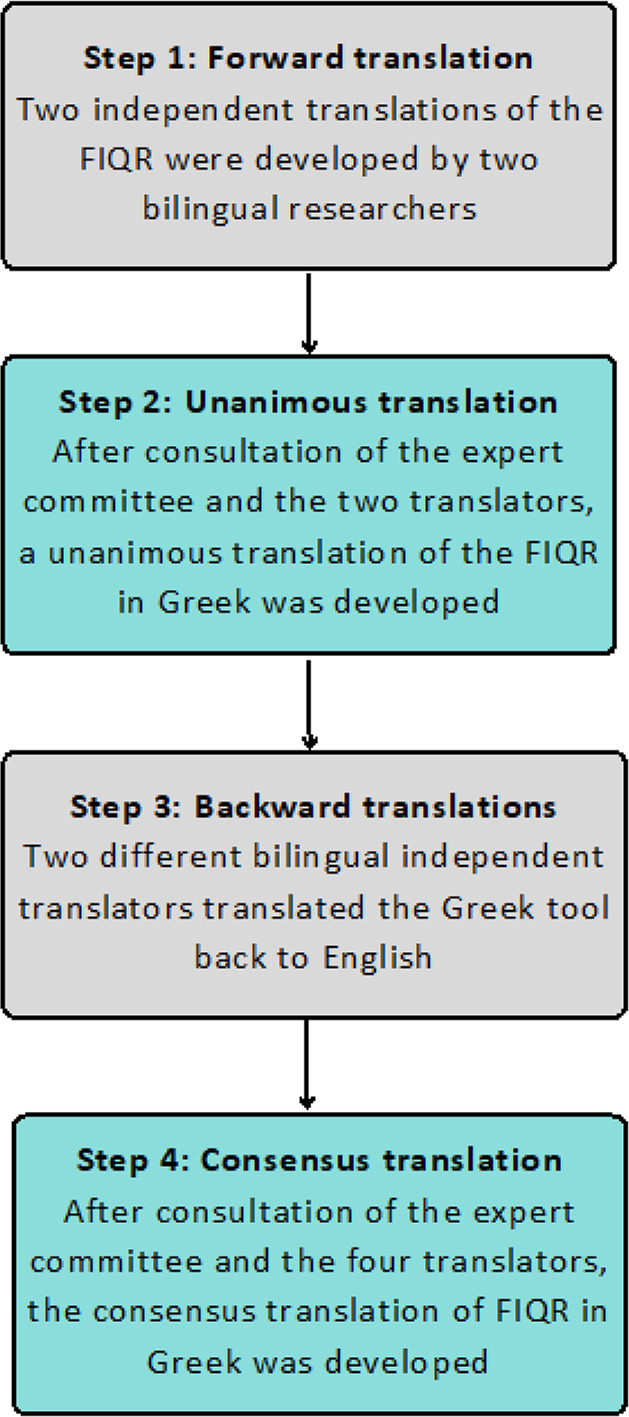
Flowchart of the translation process.

The first step is forward translation. Two bilingual translators (MGG and ECP) independently translated the original questionnaire into Greek. Each translator documented concerns related to wording or possible deviations from the original questionnaire. The second step was synthesising the translated versions, where the two versions were reviewed in a meeting with both translators (MGG and ECP) and the expert committee (DPB and DGG). Differences were resolved through discussion, leading to a unified version (FIQR-GR-preliminary). The third step was backward translation, in which two experienced English speakers (AG and EP) independently translated the FIQR-GR back into English. Each translator noted any unclear or potentially confusing items that might have altered the original meaning of the items. Finally, the expert committee, along with four translators (MGG, ECP, AG, and EP) reviewed the translated versions and discussed discrepancies and the rationale for specific word usage, producing the final version of the Greek FIQR (FIQR-GR). Additional details are provided in **Supplementary Figure 1**.

### Sample recruitment

According to the rule of thumb, at least 10 participants are required for each domain examined in a questionnaire.^21^ The FIQR has 3 domains, thus more than 30 participants were required for its validation. To increase recruitment power, patients were recruited through two routes, one being online (via patient forums) and the second being the Department of Rheumatology and Clinical Immunology in Larissa, Greece, in collaboration with private practices. All patients had been diagnosed with fibromyalgia by specialists based on the ACR 2010 criteria.^5^ Patients were included if they were adults, presented sufficient skills in reading and understanding the Greek language, and provided informed consent. The FIQR-GR was made available online from 09/2024 until 12/2024 and distributed to the participants either in person, via phone with the researcher or via a secure link. All participants were asked to provide online consent after reading the details concerning the study and the time required to complete the questionnaire. Ethical permission for the study was granted by the University Hospital of Larissa (4/16/08-11-22). All participants completed the questionnaire in full, as all fields were mandatory. In case of inquiries participants could contact a researcher via phone or email as a healthcare professional was always available to address any concerns. Apart from the FIQR domains, data regarding sociodemographic factors were collected, namely age, education, professional status, gender, weight, height, number of children and disease duration (years since diagnosis). The Body Mass Index (BMI) of each patient was calculated based on their reported height and body weight.^22^ Patients were categorised into the following groups: underweight (BMI < 18.5 kg/m^2^), normoweight (18.5 ≤ BMI < 25 kg/m^2^), overweight (25 ≤ BMI < 30 kg/m^2^), and obese (BMI ≥ 30 kg/m^2^). A total of 313 responses were collected, and the data of two participants were omitted (missing variables), leading to a final sample of 311 participants. The baseline characteristics of the patients are shown in **Table 1**. The recruitment reflected typical fibromyalgia demographics similar with the original FIQR,^11^ consisting of mostly women.

**Table 1.** Baseline characteristics of our sample.

**Variable**		**Total (N=311)**
Age (years)		48.9 ± 9.1
Body Weight (kg)		75.0 ± 16.9 (72, 63–85)
Height (cm)		165.0 ± 6.7 (165, 160–168)
BMI		27.6 ± 5.6 (26.7, 23.6–30.9)
Gender (F/M)		96.1% / 3.9% (n=299/12)
Education	Secondary education	30.2% (n=94)
	Technical school	10.9% (n=34)
	VET	12.2% (n=38)
	University	24.8% (n=77)
	Masters	20.3% (n=63)
	PhD	1.6% (n=5)
Parity	Yes	74.0% (n=230)
	No	26.0% (n=81)
Working status	Unemployed	18.6% (n=58)
	Student	1.9% (n=6)
	Private sector	32.2% (n=100)
	Public sector	27.0% (n=84)
	Freelance	12.2% (n=38)
	Pension	8.0% (n=25)
Years of diagnosis	0–5 years	53.1% (n=165)
	6–10 years	27.3% (n=85)
	11–15 years	4.5% (n=14)
	More than 15 years	15.1% (n=47)

Mean ± standard deviation was calculated for normally distributed continuous values, and median and interquartile ranges are reported in parentheses for non-normally distributed continuous values.

BMI: Body mass index; F: Female; M: Male; n: number; PhD: Doctor of philosophy; VET: Vocational education and training.

### Statistical analyses

Descriptive statistics were calculated for all demographic variables. For continuous variables, means and standard deviations (SD) were reported when data were normally distributed; otherwise, medians and interquartile ranges (IQR; 25th–75th percentiles) were provided. Categorical variables, including binary variables, were summarised using frequencies and corresponding percentages. The normality of continuous variables was assessed using the Kolmogorov–Smirnov and Shapiro–Wilk tests. For between-group comparisons, analysis of variance (ANOVA) and Student’s t-test were used for normally distributed data, while the Kruskal–Wallis test or Mann–Whitney U test was applied for non-normally distributed variables. When significant differences were detected, pairwise comparisons were performed using the Wilcoxon rank-sum test with Bonferroni correction for multiple comparisons. Correlations among continuous variables were assessed using Spearman’s rho (ρ) coefficient. Additionally, linear regression analyses were conducted to examine the associations between FIQR score, age, and BMI. Statistical analyses were performed using R Studio software [version 4.3.2 (2023-10-31), R Foundation for Statistical Computing, Vienna, Austria]. Statistical significance was set at p < 0.05.

To evaluate the internal consistency of the FIQR, reliability analyses were performed for each of its three domains: Function (9 items), Overall Impact (2 items) and Symptoms (10 items). The psych package was used to compute Cronbach’s alpha coefficients. In addition to the overall alpha values for each domain, corrected item-total correlations (*r.drop*) and Cronbach’s alpha if an item was deleted for each domain were examined, providing insight into how well each item aligns with the other items in its domain and whether any item negatively affects internal consistency. As the Overall Impact domain included two items, Cronbach’s alpha was not reported for this domain if the item was deleted, as deleting each item would render this metric meaningless. Cronbach’s alpha >0.7 is deemed satisfactory.^23^ Furthermore, we performed a confirmatory factor analysis (CFA) using the *lavaan* package according to the three factor structures proposed in previous studies.^11,14^ To evaluate the model’s goodness of fit, the Tucker Lewis Index (TLI) was calculated, providing insight into how well the hypothesised model fits the observed data, with values closer to 1 indicating a better fit.^24^ Additional goodness of fit indices included x2/ df (cut-off ≤3), comparative fit index (CFI) with values ≥0.90 deemed acceptable, root mean square error of approximation (RMSEA) with values ≤0.08 considered acceptable and standardised root mean square residual (SRMR) with values ≤0.08 considered acceptable.^25^

## RESULTS

### Comparison and correlation of FIQR scores with study variables

The translation of the FIQR into the Greek language (FIQR-GR) is provided in Open System Framework (OSF) via an e-link (https://shorturl.at/CvPGw). Overall, the participants mean age was 48.9 ± 9.1 years old, with the vast majority of the participants being women (96.1%). Most patients had completed higher education, held a university (24.8%) or a master’s degree (20.3%), and were currently working (32.2% in the private sector, 27.0% in the public sector, and 12.2% freelance). Most of the sample had children (74.0%) and had been recently diagnosed (53.1%, 0–5 years since diagnosis). The mean total score in FIQR was 65.5 ± 17.7.

FIQR scores among the participant groups according to demographic and disease characteristics are presented in **Table 2**. No differences were noted in education, working status, sex, and years since diagnosis. Different FIQR scores were noted in distinct BMI tiers (p<0.001). Pairwise comparisons showed that patients with obesity had significantly higher FIQR scores than their normoweight (p<0.001) or underweight (p=0.034) counterparts.

**Table 2.** FIQR scores among different participant groups.

**Categories**		**FIQR score**	**p-value**
Education	Secondary (*n* =94 )	68.2 ± 15.7 (71.7, 57.1–80.9)	0.122*
	Tertiary (*n* =149 )	65.5 ± 18.1 (67.5, 52.7–80.7)	
Work status	Postgraduate (*n* =68 )	61.6 ± 18.9 (63.8, 52.8–76.9)	0.072*
	Unemployed (*n* =58 )	71.1 ± 14.8 (72.9, 61–82.3)	
	Employed (*n* =222 )	63.9 ± 18.2 (66.3, 51.6–79.5)	
	Student (*n* =6 )	65.0 ± 21.9 (66.3, 56.3–79.8)	
	Retired (*n* =25 )	66.5 ± 17.1 (66.3, 57.0–77.8)	
Gender	Female (*n* =299 )	65.8 ± 17.5 (67.8, 54.2–80.4)	0.144**
	Male (*n* =12 )	57.0 ± 20.6 (63.9, 42.4–74.5)	
Years since diagnosis	0–5 years (*n* =165 )	65.1 ± 18.2 (66.3, 53.0–81.0)	0.454*
	6–10 years (*n* =85 )	64.3 ± 17.8 (67.5, 52.7–76.7)	
	11–15 years (*n* =14 )	63.3 ± 20.3 (66.8, 51.0–77.2)	
BMI	More than 15 years (*n* =47 )	69.4 ± 15 (71.8, 60.2–80.8)	<0.001*
	Underweight (*n* =5 )	45.2 ± 19.3 (37.5, 31.5–60.8)	
	Normoweight (*n* =111 )	60.8 ± 17.7 (62.2, 49.9–75.0)	
	Overweight (*n* =104 )	65.9 ± 18 (69.6, 54.3–80.3)	
	Obese (*n*=91 )	71.7 ± 14.9 (74.0, 61.8–82.8)	

FIQR is reported as mean ± standard deviation (Median, interquartile ranges)

*: Kruskal-Wallis test,

**: Mann-Whitney U test

BMI: Body mass index; FIQR: Revised fibromyalgia impact questionnaire; n: number.

Univariate linear regression analyses revealed that both BMI (β=0.86, p<0.001) and age (β=0.24, p=0.033) were significant positive predictors of the FIQR scores. This indicates that higher BMI and older age were independently associated with increased symptom severity and functional impact, as measured by FIQR. In the multiple regression model including both variables, only BMI remained a significant predictor (β=0.82, p<0.001), whereas age was no longer significant (p=0.204). This suggests that, when controlling for age, BMI is the primary factor influencing FIQR scores, while the effect of age becomes non-significant in the presence of BMI. Correlation analysis further supported these findings. A moderate positive correlation between the FIQR and BMI was observed (ρ=0.267, p<0.001), indicating that a higher BMI was associated with higher FIQR scores and a more severe fibromyalgia impact. In contrast, the Spearman correlation between FIQR and age revealed a weak, non-significant positive association (ρ=0.11, p=0.055).

### Internal consistency

The total FIQR demonstrated excellent internal consistency (Cronbach’s α=0.94), whereas all FIQR domains exhibited excellent to very good internal consistency. The *Function domain* showed excellent reliability (Cronbach’s α=0.92), while the *Overall Impact* and the *Symptoms domains* exhibited very good internal consistency (Cronbach α=0.85 in both domains).

In the *Function domain*, corrected item-total correlations ranged from 0.58 (brush or comb your hair) to 0.79 (prepare a homemade meal, change bed sheets), all within acceptable or strong ranges (**Table 3**). Cronbach’s alpha remained above 0.91 for all items when deleted, showing that no item weakened internal consistency. In the Overall Impact domain, the two items (“Fibromyalgia prevented me from accomplishing goals for the week” and “I was completely overwhelmed by my fibromyalgia symptoms”) were strongly correlated (ρ=0.75), while alphas, if the domain was omitted, were not reported because they were not interpretable for two-item scales. The *Symptoms domain* included corrected item-total correlations ranging from 0.38 (energy level) to 0.68 (pain level), indicating moderate to strong associations between each item and total domain score. When each item was removed, Cronbach’s alpha values ranged from 0.83 to 0.86, indicating that the overall internal consistency remained stable despite slight variations in individual item contributions.

**Table 3.** Corrected item-total correlations and Cronbach alphas if item is deleted.

**Domain**	**Item**	**r.drop**	**Alpha if item is deleted**
Function	1. brush or comb your hair	0.58	0.92
	2. walk continuously for 20 min	0.77	0.91
	3. prepare a homemade meal	0.79	0.91
	4. vacuum, scrub or sweep floors	0.76	0.91
	5. lift and carry a bag full of groceries	0.74	0.91
	6. climb one flight of stairs	0.71	0.91
	7. change bed sheets	0.79	0.91
	8. sit in a chair for 45 min	0.60	0.92
	9. go shopping for groceries	0.78	0.91
Overall Impact	10. fibromyalgia prevented me from accomplishing goals for the week	0.75	-
	11. I was completely overwhelmed by my fibromyalgia symptoms	0.75	-
Symptoms	12. please rate your level of pain	0.68	0.83
	13. please rate your level of energy	0.38	0.86
	14. please rate your level of stiffness	0.62	0.83
	15. please rate the quality of your sleep	0.54	0.84
	16. please rate your level of depression	0.49	0.85
	17. please rate your level of memory problems	0.56	0.84
	18. please rate your level of anxiety	0.53	0.84
	19. please rate your level of tenderness to touch	0.63	0.83
	20. please rate your level of balance problems	0.62	0.83
	21. please rate your level of sensitivity to loud noises, bright lights, odors and cold	0.55	0.84

r.drop: corrected item-total correlation.

### Confirmatory factor analysis (CFA)

CFA demonstrated goodness of fit (x^2^/df=2.63, CFI=0.900, TLI=0.887, RMSEA=0.080, SRMR=0.056). The factor loadings for most items ranged from 0.51 to 0.86, indicating that they effectively defined the latent variables, except for energy level and depression.

**Figure 1.**
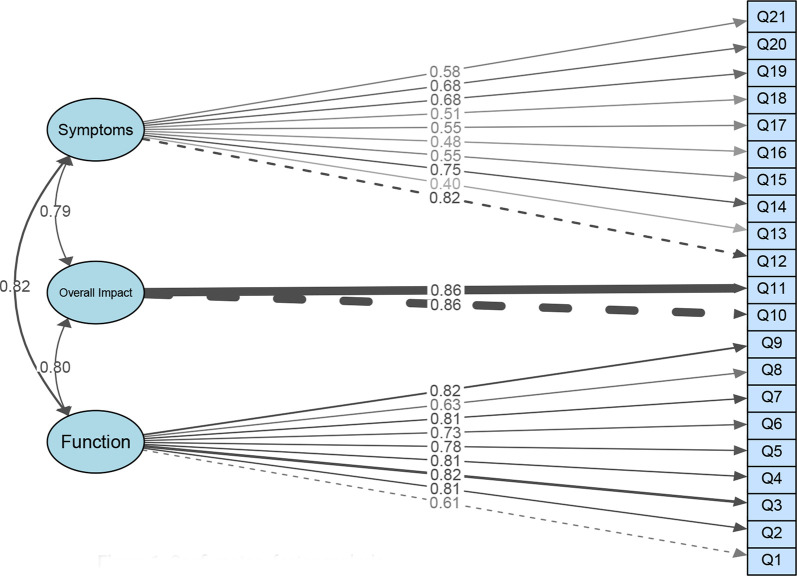
Confirmatory factor analysis.

In the *Function domain,* factor loadings ranged from 0.606 to 0.824, indicating strong associations, while in the *Overall Impact* domain, factor loadings had values of 0.862 and 0.864, reflecting very strong relationships with the latent construct. In the Symptoms domain, most items had acceptable loadings ranging from 0.508 to 0.822, with the exception of two items, namely energy levels (0.398) and depression (0.477). This suggests that these items contributed less to the latent construct (**Figure 1**).

## DISCUSSION

The present study is the first to translate and validate the FIQR in the Greek population. The findings suggest that the FIQR-GR is a reliable and valid tool for use in Greek patients with fibromyalgia. The translation process posed no substantial challenges and did not require any cross-cultural adaptations, while the questions were easy to answer and understandable to our patients.

Translations of the FIQR in other languages resulted in a Cronbach’s alpha of 0.90 in Chinese,^14^ 0.91 in Spanish and Arabic,^13,16^ 0.95 in Danish,^4^ and 0.96 in Brazilian Portuguese.^15^ The Greek version of the FIQR demonstrated excellent internal consistency with a Cronbach’s alpha of 0.94, which is consistent with the aforementioned studies. The *Function domain* showed an alpha of 0.92, comparable to the Nepali version (Cronbach’s alpha=0.90),^17^ while the *Overall Impact* and *Symptoms domains* also demonstrated good internal consistency (Cronbach’s alpha=0.85), despite the slightly lower total-item correlations of one item (energy level).

The CFA supported the original three-factor model that Bennett et al utilised^11^ with the latent variables representing Function, Overall Impact and Symptoms. Most standardised factor loadings exceeded 0.50, providing strong evidence of validity. Exceptions were energy levels and depression (0.40 and 0.48, respectively). Our findings suggest that these two items may contribute less consistently to the latent construct of Symptoms. This pattern has also been observed in other language validations, for example in the Danish version energy levels demonstrated poor reliability.^4^ This could potentially reflect the multidimensional nature of energy across different cultures. Nevertheless, the overall internal consistency of Symptoms remained high supporting the scale’s structural integrity.

Higher FIQR scores were observed among obese patients in comparison with their normoweight and underweight counterparts, a trend that is consistent with previous studies.^26^ Linear regression analyses revealed that BMI was a significant predictor of FIQR, suggesting that body weight plays an important role in fibromyalgia severity. Atzeni et al*.*^27^ demonstrated that obesity or overweight leads to greater impairment and constitutes a significant comorbidity. Obesity is a rising but modifiable global health concern that leads to a series of complications, including rheumatic diseases. Aerobic exercise for more than 20 minutes, once daily, two to three times per week, is recommended by the European League Against Rheumatism (EULAR) as an important non-pharmacological treatment for fibromyalgia.^28^ Given the negative association between obesity and fibromyalgia symptoms, body weight loss programs could facilitate a therapeutic benefit. A randomised clinical trial in patients with fibromyalgia showed that weight loss led to not only improved quality of life but additionally to reduced inflammation markers.^29^ The pathophysiology of fibromyalgia is not well understood and includes a complex network of interactions that lead to augmentation of pain perception.^30^

Despite not being an inflammatory disease *per se*, emerging data connect fibromyalgia with immune system dysregulation,^31^ with studies suggesting cytokine involvement in nociception augmentation and central sensitisation.^32,33^ Recently, proteomic studies^34^ in adult patients with fibromyalgia revealed dysregulation not only in pro-inflammatory cytokines, but also in complement and coagulation cascades and iron metabolism.^31,34–39^ One possible explanation connecting obesity and fibromyalgia is the interplay between mechanical overload and nociception. In many individuals with fibromyalgia, peripheral nociceptive inputs often arising from a co-existing inflammatory rheumatic disease can lead to disproportionate pain.^40^ In this framework, obesity can act as a mechanical stressor that triggers pain through a bottom-up process in predisposed individuals. Additionally, obesity leads to changes in muscles and tendons, potentially creating a structural substrate conducive to pain.^40^ Adipocytes found in the fat tissue secrete monocyte chemoattractant protein-1 (MCP-1), which drives macrophage M1 phenotype differentiation, producing pro-inflammatory cytokines (TNF, IL-1b, and IL-6),^41^ which are known to sensitise nociceptive receptors.^40^ While these pathways underline the complex interplay between obesity and fibromyalgia, the association is not clearly understood; therefore, current clinical practice relies heavily on diagnostic criteria and tools, such as the FIQR.

The present study included a representative sample of Greek patients, which is consistent with fibromyalgia demographics. However, the study’s limitations include the reliance on self-reported and unmeasured anthropometric parameters due to the questionnaire’s online distribution. Additionally, the cross-sectional design precludes the determination of causation in the observed associations.

## CONCLUSIONS

The Greek version of the FIQR is a reliable, validated, and easy-to-use tool both in everyday clinical settings and in clinical trials. Thus, it can be easily adapted by Greek healthcare professionals dedicated to providing care for patients with fibromyalgia.
